# Case of Presentation of the Head and Foot

**Published:** 1844-09-07

**Authors:** Hattersly P. Worthington

**Affiliations:** Elkridge Landing, Maryland


### CASE OF PRESENTATION OF THE HEAD AND FOOT,

By Hattersly P. Worthington, M. D.
Elkridge Landing, Maryland.

February 4th, 1844, I was called to attend Mrs. W., in labour with her first child. My patient was tall, slender, though of a good constitution; her age over thirty. When I first saw her, labour was just commencing; os tincæ slightly dilated, the parts relaxing. The pelvis was well formed, and of medium size. The labor advanced gradually, the head presenting, and all promised a safe delivery. After six or eight hours the *os tincæ* was fully dilated, and the head well engaged in the inferior strait;—the pains were strong, regular and frequent, but the head did not advance proportionately, and my patient's strength began to fail. She was becoming more and more exhausted, when, with one prolonged effort, the head passed into the world, and I detected the left foot lying by the side of the right ear. With another pain, the foot was retracted and the child born. The position of the foot had disfigured the head, distorting the lower jaw to the opposite side. The child lived, and has continued in good health. The disfigurement has entirely disappeared.

This presentation is spoken of by but few writers. Dr. Lee mentions it as an extremely rare case. It is one in which little assistance can be given by art, for its nature renders it uncertain until the head is born. Could it be detected at the commencement of, or early in the labour, the indication would undoubtedly be, if possible, to disengage and return the foot, or else to turn and deliver by the feet; for I cannot suppose that most cases would naturally terminate as favorably, to mother and child, as the one I have reported.

August 19th, 1844.

